# microRNA-352 regulates collateral vessel growth induced by elevated fluid shear stress in the rat hind limb

**DOI:** 10.1038/s41598-017-06910-9

**Published:** 2017-07-27

**Authors:** Yinglu Guan, Baizhen Cai, Xiaoqiong Wu, Song Peng, Liaoying Gan, Da Huang, Guangmin Liu, Liping Dong, Lin Xiao, Junwen Liu, Bin Zhang, Wei-Jun Cai, Jutta Schaper, Wolfgang Schaper

**Affiliations:** 1 Department of Histology & Embryology, School of Basic Medicine, Central South University Changsha, 410013 Hunan, P.R. China; 2 Department of Intensive Care Unit, the 3rd Xiangya Hospital, Central South University Changsha, 410013 Hunan, P.R. China; 3 Department of Anatomy & Neurobiology, School of Basic Medicine, Central South University Changsha, 410013 Hunan, P.R. China; 4 Department of Radiology, the 3rd Xiangya Hospital, Central South University Changsha, 410078 Hunan, P.R. China; 5 Max-Planck-Institute for Heart and Lung Research, Arteriogenesis Research Group, Bad Nauheim, D-61231 Germany; 6 Department of Pharmacological & Pharmaceutical Sciences, College of Pharmacy, University of Houston, 77204 Houston, TX United States

## Abstract

Although collateral vessel growth is distinctly enhanced by elevated fluid shear stress (FSS), the underlying regulatory mechanism of this process remains incompletely understood. Recent studies have shown that microRNAs (miRNAs) play a pivotal role in vascular development, homeostasis and a variety of diseases. Therefore, this study was designed to identify miRNAs involved in elevated FSS-induced collateral vessel growth in rat hind limbs. A side-to-side arteriovenous (AV) shunt was created between the distal stump of one of the bilaterally occluded femoral arteries and the accompanying vein. The miRNA array profile showed 94 differentially expressed miRNAs in FSS-stressed collaterals including miRNA-352 which was down-regulated. Infusion of antagomir-352 increased the number and proliferation of collateral vessels and promoted collateral flow restoration in a model of rat hind limb ligation. In cell culture studies, the miR-352 inhibitor increased endothelial proliferation, migration and tube formation. In addition, antagomir-352 up-regulated the expression of insulin-like growth factor II receptor (IGF2R), which may play a part in the complex pathway leading to arterial growth. We conclude that enhanced collateral vessel growth is controlled by miRNAs, among which miR-352 is a novel candidate that negatively regulates arteriogenesis, meriting additional studies to unravel the pathways leading to improved collateral circulation.

## Introduction

Under ischaemic conditions, collateral vessel growth or arteriogenesis (from pre-existing interconnecting arterioles to functional collateral arteries) is the body’s natural repair mechanism to restore blood flow to ischaemic tissues. The formation of functional collateral arteries can enhance the perfusion of ischaemic tissues approximately 10-fold^1^. Therefore, induction of arteriogenic reactions to enhance collateral vessel growth, also called therapeutic arteriogenesis has been suggested as a promising approach for the treatment of severe vascular occlusive disease^1^. In recent decades, dozens of arteriogenic factors, including FGFs, VEGF, PDGF and monocytes, have been studied with varying success in various experimental models and in small open trials for the treatment of patients with coronary and periphery arterial diseases. However, the data from two double-blinded, randomized, and placebo controlled clinical trials failed to provide evidence of its efficiency in the treatment of ischaemic patients^2^, indicating that a better understanding the molecular mechanisms of arteriogenesis and additional novel arteriogenic factors are required for the development of therapeutic arteriogenesis.

Fluid shear stress (FSS) has been acknowledged as a key trigger of collateral vessel growth. Once chronic occlusion or stenosis becomes significant, blood flow is directed towards the region with the lowest resistance via the pre-existent arterioles that now connect a high-pressure region with a low-pressure region^3^; this results in increased shear stress, which modulates many physiological, biochemical, and molecular responses in *in vitro* and *in vivo* experiments^4–6^. Indeed, we recently showed that elevated FSS achieved through an arteriovenous shunt (AV-shunt) created between the distal stump of an occluded femoral artery with the accompanying vein markedly amplifies collateral vessel growth^7^. Furthermore, increased expression of several biologically active substances, such as vascular endothelial cell adhesion molecules, inflammatory factors and growth factors was evident and several groups of differential mRNAs have been identified using this AV-shunt model^8, 9^. These data strongly suggest that increased FSS induced by arterial occlusion controls collateral vessel growth via regulating gene expression at both the protein and mRNA levels.

microRNAs (miRNAs) are a novel class of regulatory noncoding, conserved, single-strand RNAs that negatively regulate over 30% of gene expression at the posttranscriptional level by binding to 3′ untranslated regions (3′-UTR) of target mRNAs^10, 11^. In the past few years, increasing evidence has documented an important regulatory function of miRNAs in angiogenesis^12–14^. More recently, several studies have demonstrated that a different miRNA expression profile is induced in collateral vessels of mice following femoral artery occlusion, and several miRNAs such as miR-100, miR-155, miR-329 and miR-495 were found to be important factors that control collateral vessel growth^15–17^. These data highlight the importance of studies on the role of miRNA in regulating collateral vessel growth induced by FSS. Based on the above-mentioned findings and our previous findings that high levels of FSS lead to differential mRNA expression in collateral vessels between the AV-shunt side and the ligature-only side^9^, we hypothesized that there exists novel miRNAs that could play an important role in amplifying collateral vessel growth in response to elevated FSS.

In the present study, we demonstrated a comprehensive miRNA expression profile in growing collateral vessels in the rat AV-shunt model. Next, we identified miR-352 as the most consistently down-regulated miRNA and investigated its localization in collateral vessels as well as its functions in collateral vessel growth. We determined that miR-352 is expressed in endothelial cells and inhibits arteriogenesis *in vivo* and angiogenesis *in vitro* via regulating its target gene insulin-like growth factor II receptor/ cation-independent mannose 6-phosphate (IGF2R/CI-M6P).

## Results

### Differential expression of miRNAs in collateral vessels and the miR-352 expression pattern

**Figure 1:**
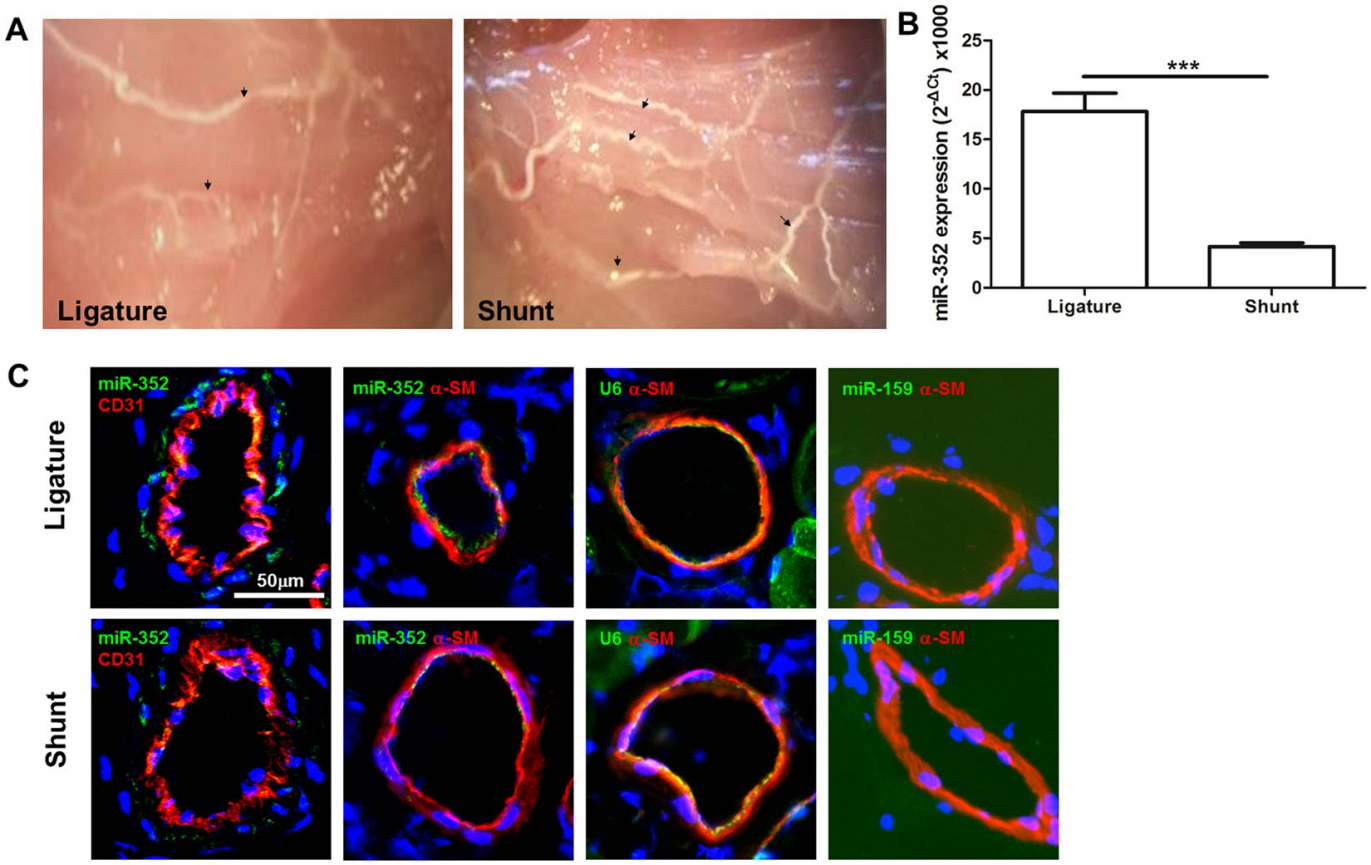
Expression of miR-352 in collateral vessels of the ligature-only side (LO) and the AV-shunt side (AVS). (**A**) Collateral vessels that were infused with liquid latex in the superficial adductor muscles from both the LO and AVS. An increased number of collaterals was evident in the AVS (indicated by arrows); original magnification: 10×. (**B**) Quantitative analysis of miR-352 expression by stem-loop qPCR in LO and AVS collateral vessels from each animal (***P < 0.001, n = 5). (**C**) *In situ* hybridization from miR-352 in collateral vessels of the LO and AVS. Green fluorescence and red fluorescence for CD31 and α-SM-actin, respectively. The U6 probe was used as a positive control, and miR-159 served as a negative control because this miRNA is not expressed in mammalian tissue. Notably, miR-352 was present mainly in endothelial and smooth muscle cells of the collateral vessels, and its expression was decreased in collateral vessels of the AVS as compared with those in the LO.

To investigate changes in the miRNA transcriptome induce by elevated FSS in collateral vessels, miRNA microarray analysis was performed with total RNA extracts of the isolated collateral vessels isolated from the superficial adductor muscles from both the ligature and AV-shunt sides (Figure S1 includes a graphic representation of the AV-shunt). An increase in the number of collateral vessels was evident in the AV-shunt side compared to the ligature side (Fig. 1A). Among 3100 capture probes on the microarrays, 94 differentially expressed miRNAs (2-fold changes) were identified, among which 44 miRNAs were up-regulated and 50 were down-regulated; they are listed in the online-only Data Supplement (Figure S2 and Table S1).

Among those differentially expressed miRNAs (2-fold changes), many have been described to be involved in the regulation of cell proliferation, migration, MMPs, apoptosis, angiogenesis and growth factors (Tables 1 and 2), which are important factors that contribute to collateral vessel growth, indicating the validity of our experimental approach. On the other hand, of those differentially expressed miRNAs, a few have not previously been described or have been rarely reported, for example, miR-352. We screened potential miRNA seed sequences in the 3′-UTR using 3 bioinformatic algorithms (miRanda [www.microrna.org], miRDB [mirdb.org/miRDB] and targetscan [www.targetscan.org]), and identified 15 genes with a miR-352 target site predicted by all three bioinformatic algorithms (Figure S4); one of them is IGF2R. IGF2R has angiogenic potency^18, 19^, and a previous study has demonstrated that IGF2R is up-regulated in collateral vessels in response to elevated FSS in a rabbit model^8^. Therefore, we speculated that miR-352 could regulate angiogenesis and arteriogenesis. To our knowledge, the only available information about the function of miR-352 was reported in Tao’s study, which showed that miR-352 is involved in the lysosomal-autophagy pathway^20^. Autophagy (“self-eating”) constitutes one of the most spectacular yet subtly regulated phenomena in cell biology. Because miR-352 has not been studied before in the context of collateral vessel growth, we selected it for further investigation. Quantitative reverse-transcriptase polymerase chain reaction (qRT-PCR) using miRNA stem loop primers for miR-352 was performed at one week after ligation or AV-shunt creation in 5 rats per group. The results of the qRT-PCR experiment showed that miR-352 expression was constant in each sample and confirmed our microarray data, which showed that miR-352 was expressed in collateral vessels on ligation side and significantly decreased in shunt side (Fig. 1B). Using *in situ* hybridization and immunofluorescence for vascular markers, including a hybridization probe against U6 as a positive control, and a hybridization probe against miR-159 was used as a negative control (because this miRNA is not expressed in rat tissue), we found that miR-352 was mainly expressed in the endothelium and tunica media of collaterals and was significantly decreased in shunt-side collateral vessels (Fig. 1C).

**Table 1:** Twenty up-regulated miRNAs.

miRNA	Fold change AV-shunt vs. control	Function
rno-miR-653-3p	37.56097561	Unknown
rno-miR-3594-3p	9.31358885	Unknown
rno-miR-483-3p	8.414634146	Anti-apoptotic oncogene, cardiovascular inflammation and remodelling
rno-miR-92b-5p	8.096126255	Heart development and atrial fibrillation
rno-miR-377-5p	4.84735544	Atrial fibrillation and structural remodelling
rno-miR-341	4.764878049	Atrial fibrillation and TGF-β/Smad2/3 pathway
rno-miR-21-3p	4.637785208	Cell proliferation and invasion.
rno-miR-675-5p	4.143757751	Premature senescence of cardiac progenitor cells, G1 arrest, reduced cell proliferation, colony formation, migration and invasion
rno-miR-183-3p	3.74730108	Regulates claudin-1 expression
rno-miR-299a-5p	3.626723224	Anti-apoptotic role
rno-miR-200c-3p	3.593610443	Targets the VEGF-VEGFR2 pathway and angiogenesis
rno-miR-665	3.511737089	Negatively targets anti-apoptotic BCL2L1
rno-miR-291a-5p	3.457928187	VSMC migration
rno-miR-490-5p	2.373358	Tumour suppressor
rno-miR-1	2.505729	Suppresses cell growth
rno-miR-133b	2.192279	Inhibits cell proliferation and invasion
rno-miR-30c-1-3p	2.70761	Suppresses PXR expression
rno-miR-294	2.010496	Promotes proliferation and differentiation
rno-miR-127-5p	2.780488	A regulator of MMP-13 and suppresses cell growth
rno-miR-503	2.327383	Inhibits cell proliferation and invasion

**Table 2:** Twenty down-regulated miRNAs.

miRNA	Fold change AV-shunt vs. control	Function
rno-let-7f-5p	−5.200726271	Proangiogenesis and pulmonary arterial hypertension
rno-miR-98-5p	−4.790951973	Inhibits angiogenesis and regulates MMP-11
rno-miR-100-5p	−4.56496786	Neovascularization
rno-miR-30a	−2.712938546	Inhibits the proliferation and migration of endothelial cells, promotes apoptosis and reduces autophagy activity
rno-let-7a-5p	−3.643793535	Inhibits endothelial and vascular smooth muscle cell proliferation
rno-miR-26a-5p	−3.478532104	Suppresses angiogenesis
rno-miR-99a-5p	−3.150019751	Suppresses proliferation and migration
rno-miR-497-5p	−2.888349061	Anti-angiogenesis and negatively regulates MMP-9 and VEGFA
rno-miR-352	−2.873047456	Regulates lysosomal-associated membrane protein 2
rno-let-7d-5p	−2.854490731	Vascular smooth muscle cell proliferation
rno-miR-29b-3p	−2.78013317	Atypical coronary artery disease, abdominal aortic aneurysm suppresses angiogenesis and regulates MMP-2 and VEGF
rno-miR-30c-5p	−2.774625087	Angiogenesis, vascular smooth muscle cell calcification, regulates angiogenesis and autophagy
rno-let-7c-5p	−2.738042055	let-7 family and inhibits proliferation and migration
rno-miR-145-5p	−2.209690820	Inhibits angiogenesis
rno-miR-27b	−2.373222456	Inhibits angiogenesis and regulates VEGF-C and MMP-13
rno-let-7b	−2.14686718	Promotes angiogenesis and suppresses apoptosis and autophagy
rno-miR-30e	−2.15284326	Induces apoptosis and regulates SMC proliferation and differentiation
rno-miR-10a-5p	−2.36103536	Pro-angiogenic activity and regulates VEGF and VE-cadherin
rno-miR-181a	−2.1600044	Inhibits angiogenesis and regulates MMP-14
rno-miR-27a-3p	−2.6992738	Regulates VE-cadherin, cell proliferation, apoptosis and MMP13

In addition, to exclude possible contamination of muscle tissue in the transcriptome analysis, adductor muscles were harvested and used as positive controls for the RT-qPCR analysis of troponin I1 (Tnni1) and troponin I2 (Tnni2) in collateral vessels, since these two genes are specifically expressed in skeletal muscle. Expression of Tnni1 and Tnni2 was detected in the skeletal muscle but not in collaterals, where their Cq values were above 40 (Figure S3), indicating that there was no contamination by skeletal muscle in this study.

### miR-352 has anti-angiogenic potency ***in vitro***

**Figure 2:**
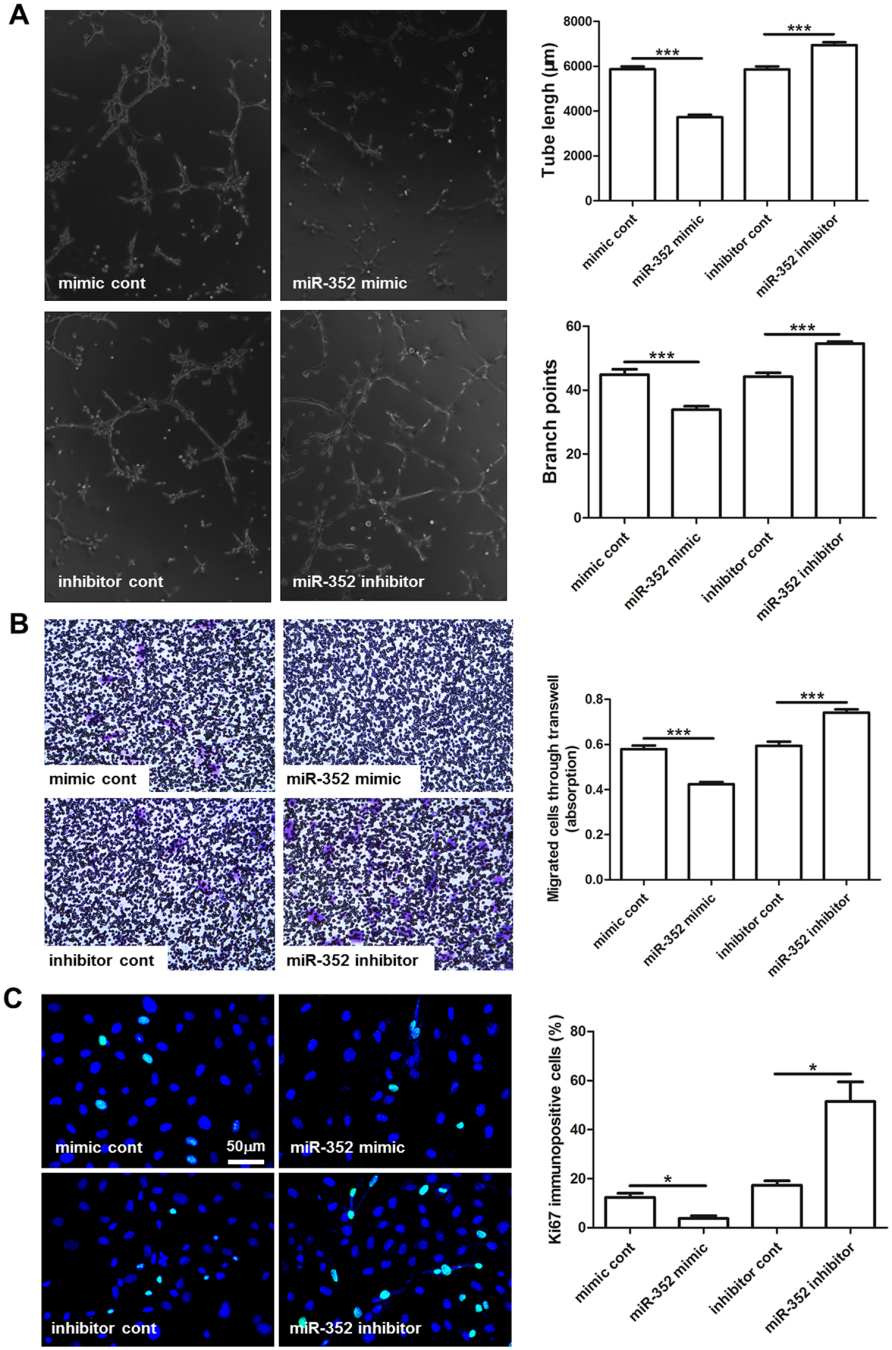
Effect of miR-352 overexpression or inhibition on angiogenesis in cultured rat aorta endothelial cells (RAECs). RAECs were transfected with either miR-352 mimic, miR-352 inhibitor or the respective negative controls: mimic control/inhibitor control. (**A**) Transfected cells were cultured on Matrigel, and the cumulative sprout length and branch points of capillary-like structures were measured after 6 hours. miR-352 mimic attenuated angiogenic tube formation, whereas inhibition of miR-352 had a stimulatory effect. (***P < 0.001, n = 3). (**B**) Stained migratied transfected cells on the bottom side of the Transwell chamber after 24 hours. miR-352 mimic attenuated cell migration, whereas inhibition of miR-352 promoted cell migration. (***P < 0.001, n = 3). (**C**) Proliferation of transfected cells was examined by Ki67 immunostaining. Green for Ki67, and blue for nuclei. miR-352 mimic attenuated cell proliferation, whereas miR-352 inhibitor promoted cell proliferation (*P < 0.05, n = 3).

To investigate the potential role of miR-352 in angiogenesis, we used miR-352 mimic and inhibitor, respectively to overexpress and inhibit miR-352 in cultured rat aorta endothelial cells. CD31 antibody was used to verify the endothelial cells, showing a positive rate of >95%, and an optimized transfection protocol using a Cy3-conjugated mimic negative control resulted in a transfection efficiency of >90% in endothelial cells (Figure S5). qPCR showed that miR-352 expression was strongly increased at 24 hours after transfection with miR-352 mimic, whereas transfection with miR-352 inhibitor resulted in a significant decrease in miR-352 expression (Figure S6). Transfection of the different compounds did not affect cell viability (Figure S7). A mimic and an inhibitor with irrelevant nucleotide sequences served as negative controls. The overexpression of miR-352 resulted in a decrease in endothelial cell network formation in the planar Matrigel (Fig. 2A), cell migration (Fig. 2B) and cell proliferation assays (Fig. 2C). In contrast, the inhibition of miR-352 resulted in an increase in endothelial cell network formation (Fig. 2A), cell migration (Fig. 2B) and cell proliferation (Fig. 2C). Taken together, we concluded that miR-352 has anti-angiogenic potency in endothelial cells.

### Inhibition of miR-352 by antagomir treatment promotes collateral vessels growth and collateral flow restoration in rat hind limbs

Based on the previous results, we tested whether inhibition of miR-352 promotes collateral vessel growth *in vivo*. miR-352 was modulated *in vivo* after femoral ligature by the additional application of antagomir-352 via osmotic mini pumps for a period of one week (Figure S8A). qPCR showed that antagomir-352 treatment resulted in a strong suppression of miR-352 expression in collateral vessels (Figure S8B).

**Figure 3:**
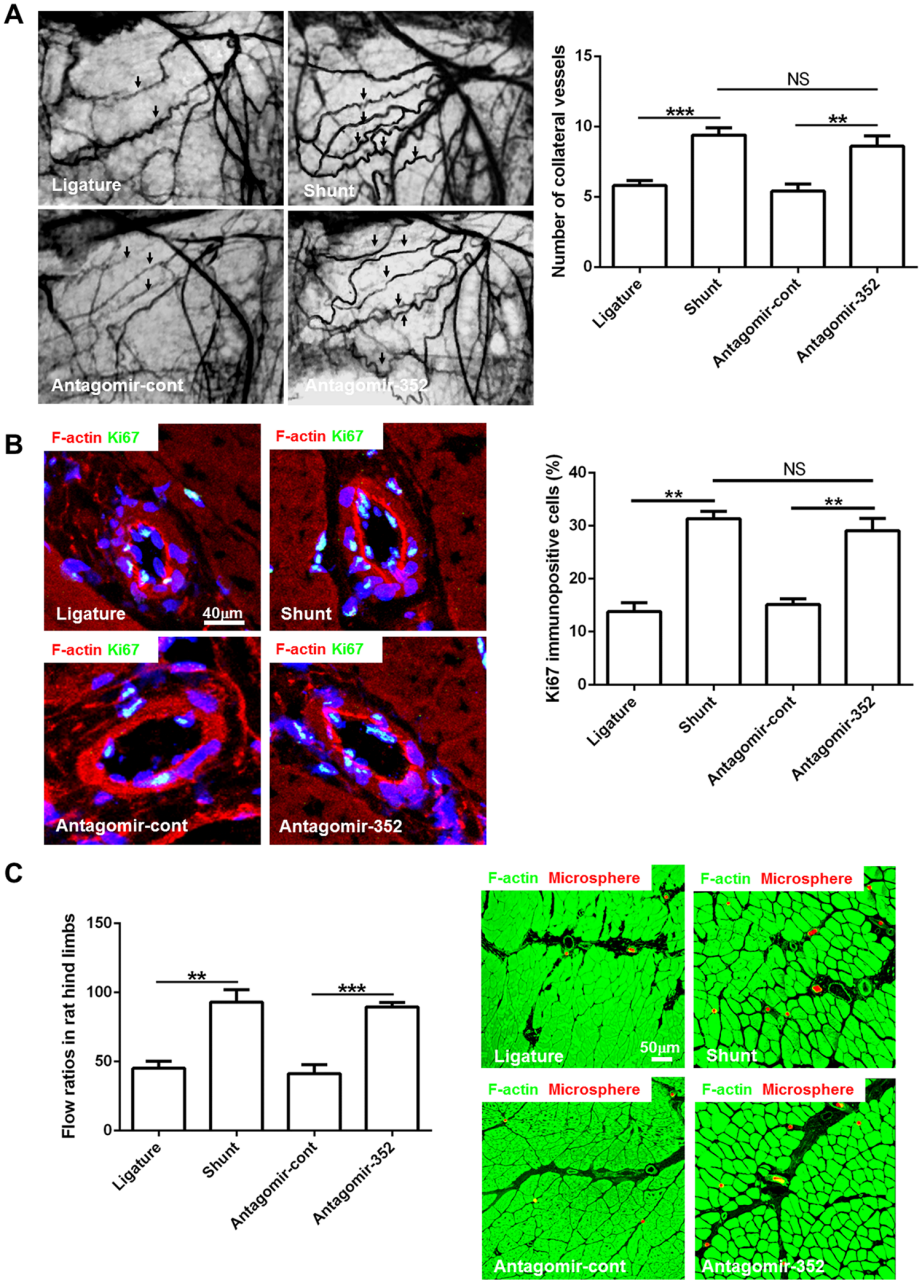
Inhibition of miR-352 enhances arteriogenesis and promotes flow restoration in rat hind limbs. Ligature: the ligature-only side; Shunt: the AV shunt side; antagomir-352: rats infused with antagomirs targeting miR-352 through an osmotic mini pump connected to the proximal region of the occluded femoral artery; and antagomir-cont: control antagomir. (**A**) Postmortem angiograms of rat hind limbs on day 7 after infusion. Antagomir-352 treatment resulted in an increase in the number of collateral vessels (arrows) as compared to antagomir-cont treatment. This increase is similar to the number of collateral vessels observed following shunt treatment. (***P < 0.001, **P < 0.01, n = 5). (**B**) Cell proliferation was detected by Ki67 immunostaining in collateral vessels from different groups. Green fluorescence for Ki67, red for F-actin, blue for nuclei. Notably, Ki67-positive cells were more numerous in collaterals treated with antagomir-352 than in the control side, which is similar to what is observed following shunt treatment (**P < 0.01, n = 5). (**C**) Flow ratio of rat hind limbs at day 7 after antagomir-352 infusion and fluorescence microspheres administration in adductor muscles. Antagomir-352 treatment resulted in a significant increase in collateral flow, compared to antagomir-cont treatment. This increase is similar to that observed following shunt treatment (**P < 0.01, ***P < 0.001, n = 5). Red fluorescence for the fluorescence microspheres, and green for F-actin. The number of fluorescence microspheres was higher in the antagomir-352-treated and AV shunt-side muscles.

To quantify arteriogenesis, the number of collateral vessels and proliferative cells in the vascular wall was determined by angiogram and Ki67 immunofluorescence staining, respectively. Analysis of the number of collateral vessels revealed a significant increase after one week of antagomir-352 treatment vs. antagomir control (Fig. 3A). In addition, Ki67 staining of collateral vessels revealed an increased proliferative activity of the vascular cells after miR-352 inhibition (Fig. 3B). It was noticed that although antagomir-352 treatment resulted in an increase in the number of collateral vessels and cell proliferation, these arteriogenic activities were still slightly less than those in shunt-treated animals (Fig. 3).

Seven days after the initial operation and antagomir treatment, flow restoration in the simple femoral ligatured hind limbs was 45.2 ± 5.0%, in the AV-shunted hind limbs was 93.1 ± 8.9%, in the femoral ligatured hind limbs treated with antagomir-352 was 89.6 ± 3.3%, and in the antagomir controls was 41.3 ± 6.4%, as compared with the non-occluded hind limbs (Fig. 3C). Analysis of flow restoration showed a significant increase after a week of antagomir-352 treatment vs. antagomir control (Fig. 3C). Histological studies of fluorescence microsphere-perfused tissues showed that the number of fluorescence microspheres was higher in the antagomir-352-treated hind limbs than in the antagomir controls (Fig. 3C).

### IGF2R is a target of miR-352

**Figure 4:**
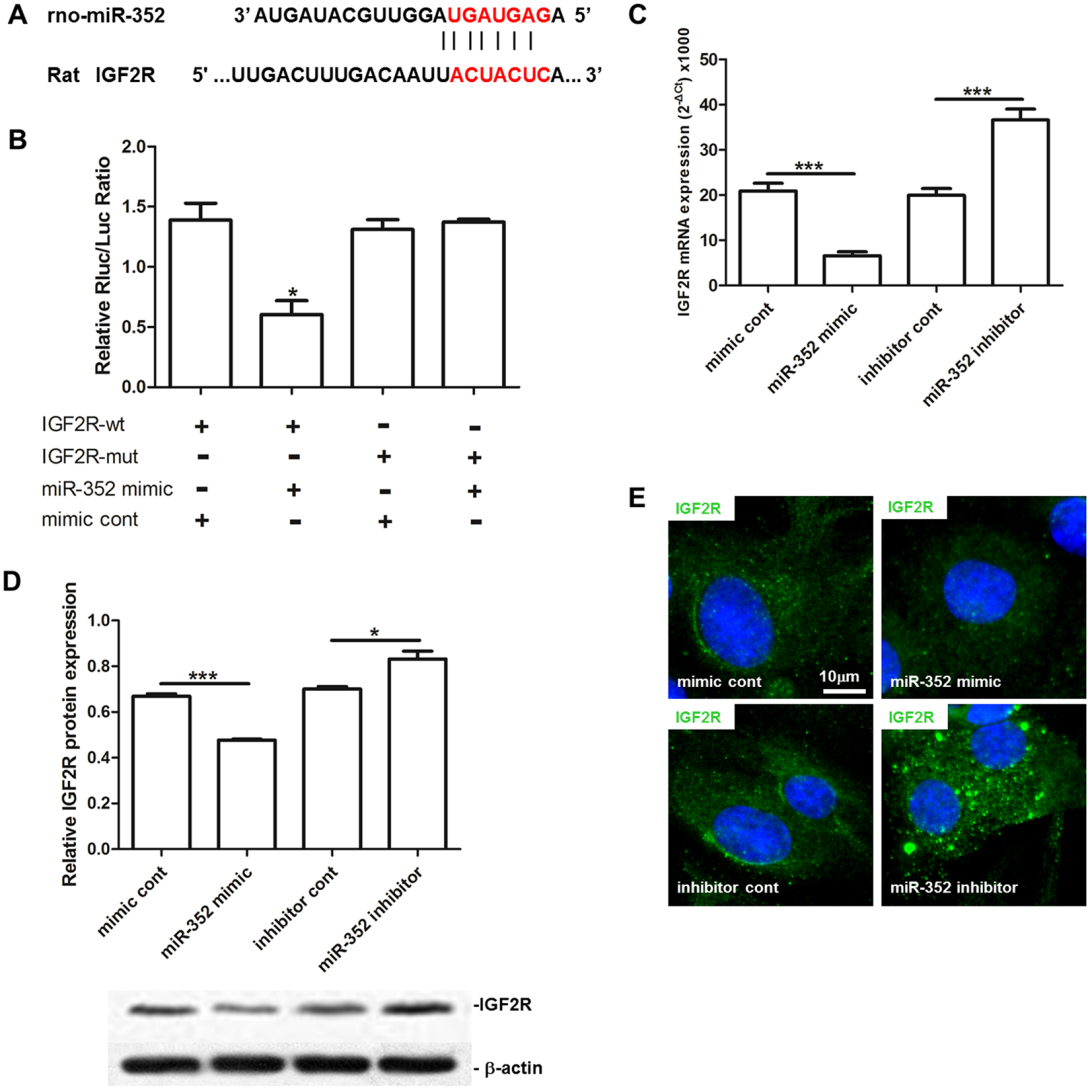
Identification of IGF2R as a target of miR-352 in rat aorta endothelial cells (RAECs). (**A**) Seed sequence of miR-352, and the complementary 3′-UTR sequence of IGF2R. (**B**) Luciferase analysis. Luciferase activity of the IGF2R wt 3′-UTR construct but not the IGF2R mut construct, was decreased in the miR-352-expressing cells (*P < 0.05, n = 3). (**C**–**E**) Overexpression or inhibition of miR-352 in RAECs resulted in a decrease or an increase in IGF2R expression on both the mRNA (**C**) and protein (**D**,**E**) levels as determined by qPCR (**C**), western blot (**D**) and immunofluorescence staining (**E**), respectively. Specific fluorescence in green, and nuclei staining in blue (***P < 0.001, *P < 0.05, n = 3).

Given that miR-352 regulates angiogenesis and arteriogenesis, that all three bioinformatics algorithms predicted that IGF2R has a binding site for miR-352 and that IGF2R was up-regulated in collateral vessels by elevated FSS in a rabbit model^8^, we considered IGF2R to be an important potential target. IGF2R contains a miR-352 binding site in the 3′-UTR of its mRNA (Fig. 4A). To test whether miR-352 directly regulates IGF2R, we used a dual-luciferase reporter assay in RAECs transfected with either wild-type or mutated IGF2R 3′-UTR firefly luciferase constructs and miR-352 mimic or negative mimic control. Transfection of miR-352 mimic inhibited luciferase activity, whereas mutant or mimic control had no effect (Fig. 4B), indicating that miR-352 can down-regulate IGF2R expression by binding to its 3′-UTR.

Furthermore, using qPCR, western blotting and immunofluorescence, we investigated the direct regulation of IGF2R by miR-352 in RAECs. Overexpression of miR-352 reduced IGF2R expression on both the mRNA and protein level in RAECs, whereas knockdown of miR-352 resulted in a significant up-regulation of IGF2R (Fig. 4C–E). These indicated that IGF2R is regulated by miR-352 in RAECs.

**Figure 5:**
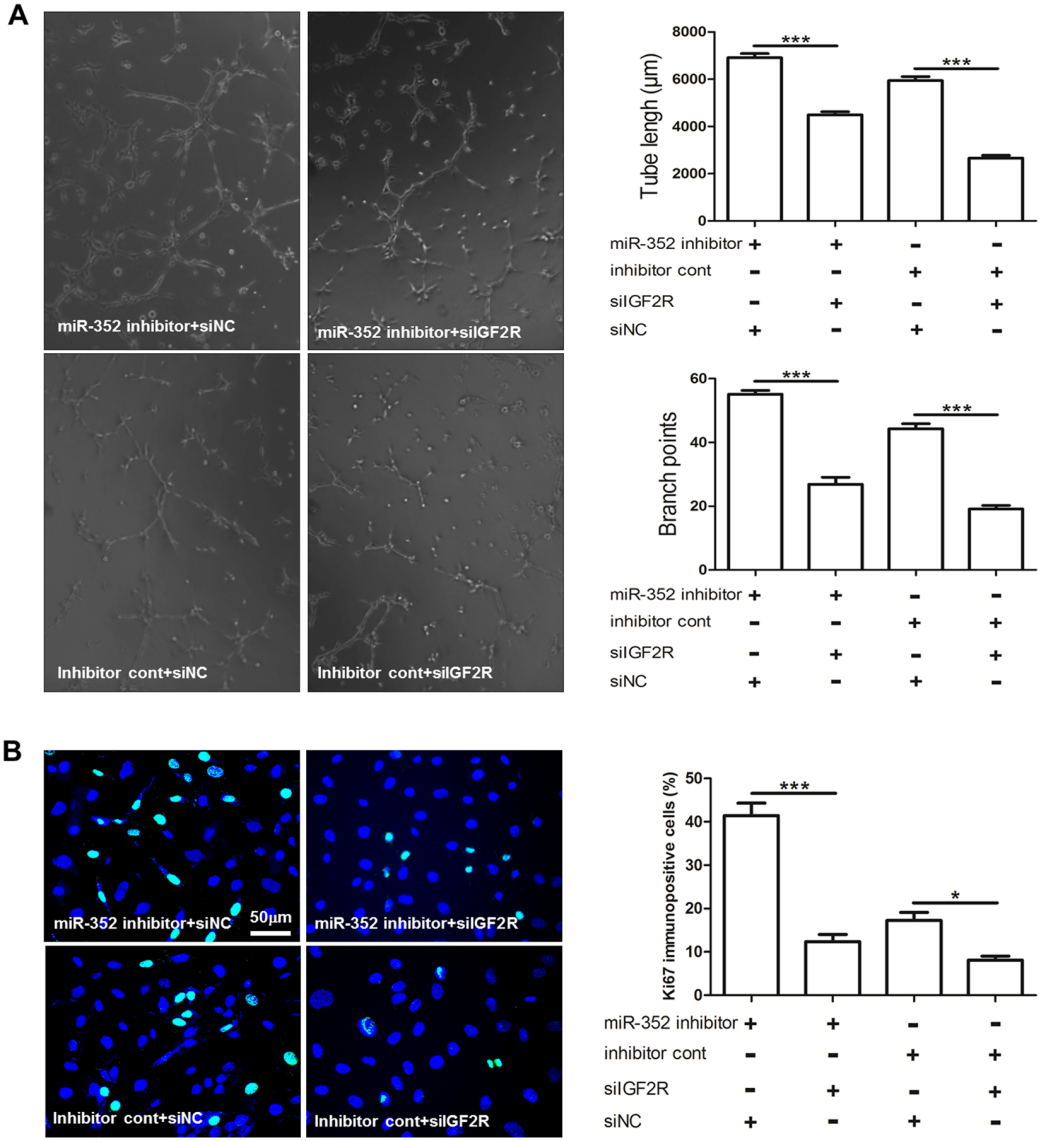
miR-352 regulates endothelial tube formation and cell proliferation in cultured RAECs by targeting the IGF2R gene. (**A**) Effect of miR-352 inhibitor + siIGF2R (IGF2R siRNA); miR-352 inhibitor + siNC (a scrambled siRNA); siIGF2R + inhibitor cont; or siNC + inhibitor cont on endothelial tube formation (***P < 0.001, n = 3). (**B**) Effect of the miR-352 inhibitor + siIGF2R (IGF2R siRNA); miR-352 inhibitor + siNC (a scrambled siRNA); siIGF2R + inhibitor cont or siNC + inhibitor cont on RAEC proliferation was detected by Ki67 immunofluorescence. Specific fluorescence in green, and nuclei are labelled in blue. Notably, treatment with miR-352 inhibitor + siIGF2R or inhibitor cont + siIGF2R significantly reduced both endothelial tube formation and cell proliferation in cultured RAECs as compared to treatment with miR-352 inhibitor + siNC or inhibitor + siNC, respectively, indicating that IGF2R is a miR-352 target in RAECs (***P < 0.001, *P < 0.05, n = 3).

To examine the biological relevance of reduced IGF2R by miR-352, we tested endothelial tube formation and cell proliferation in cultured RAECs treated with miR-353 inhibitor versus siIGF2R. qPCR showed that treatment with siIGF2R resulted in the suppression of IGF2R expression in RAECs (Figure S9). We found that treatment with miR-352 inhibitor + siIGF2R or Inhibitor cont + siIGF2R significantly reduced both endothelial tube formation and cell proliferation in cultured RAECs compared to those in RAECs treated with miR-352 inhibitor + siNC or inhibitor + siNC (Fig. 5).

### Effect of FSS and antagomir-352 on the expression of IGF2R, IGF2, Beclin1, LC3a, LC3b and ATG5 during arteriogenesis

**Figure 6:**
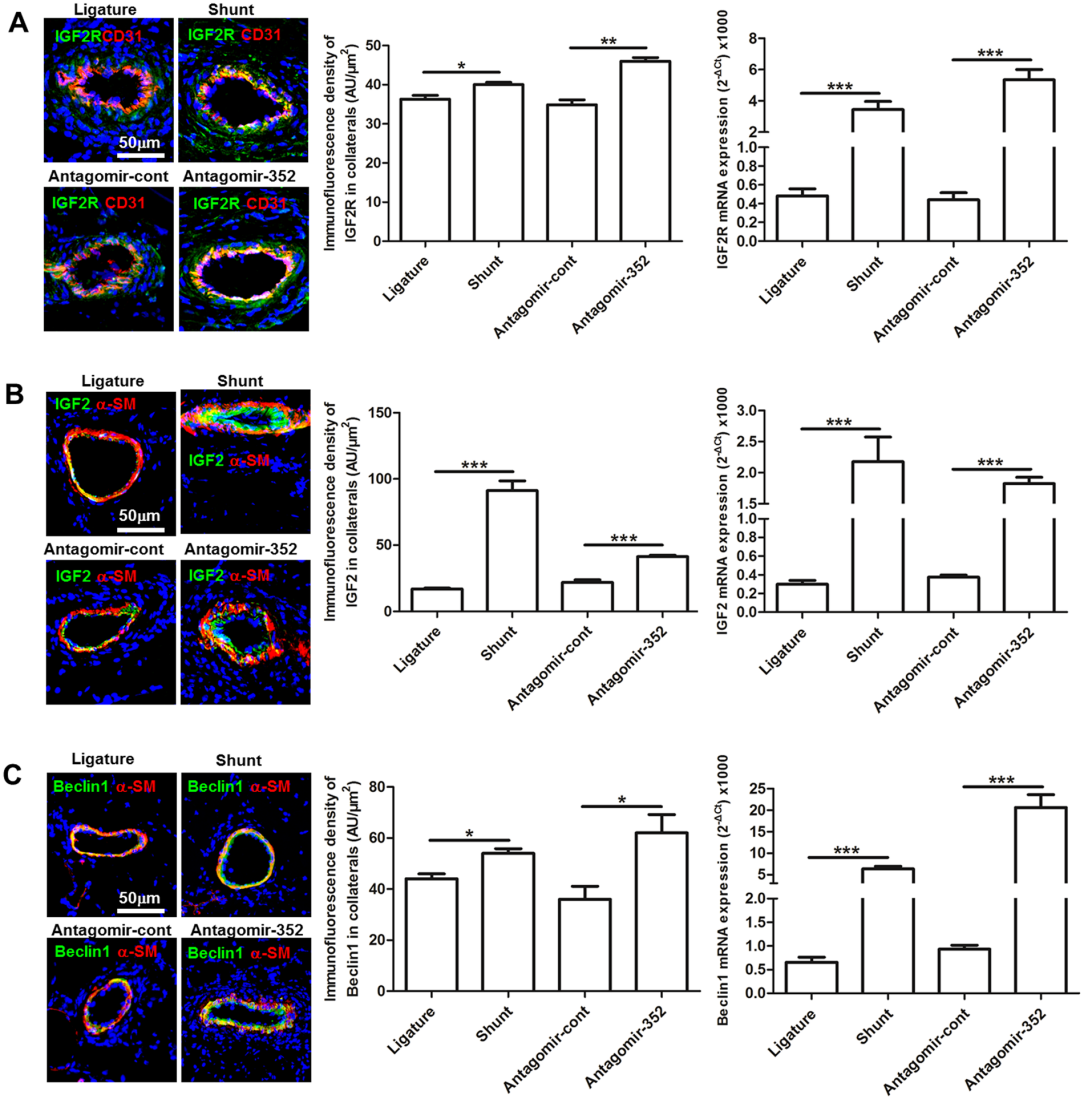
Effect of FSS and antagomir-352 on the expression of IGF2R, IGF2 and Beclin1 in collateral vessels of rat hind limbs. (**A**) IGF2R immunostaining and qPCR. (**B**) IGF2 immunostaining and qPCR. (**C**) Beclin1 immunostaining and qPCR. Specific fluorescence in green; red fluorescence for CD31 in A and for α-SM-actin in (**B** and **C**) blue fluorescence labels nuclei. Ligature: ligature-only side; Shunt: AV-shunt side; antagomir-352: antagomir-352 treated-side; antagomir-cont: antagomir-352 control. Both shunting and antagomir-352 treatment up-regulated expression of IGF2R, IGF2 and Beclin1 (***P < 0.001, **P < 0.01, *P < 0.05, n = 5).

Because we found that miR-352 regulated IGF2R expression *in vitro* and because the literature showed that miR-352 is involved in the lysosomal-autophagy pathway, our next aim was to test whether inhibition of miR-352 altered the expression of IGF2R, IGF2, Beclin1, LC3a, LC3b and ATG5 *in vivo*. As expected, FSS or antagomir-352 treatment significantly increased both the mRNA and protein levels of IGF2R as compared to femoral ligature or antagomir control rats (Fig. 6A). Similar to IGF2R expression, increased expression of IGF2, a cognate ligand of IGF2R, was observed in either FSS or antagomir-352-treated rats (Fig. 6B). Expression of autophagic markers, Beclin1, LC3a, LC3b and ATG5 was significantly increased in the collateral vessels of shunt or antagomir-352-treated rats (Figs 6C and S10).

To verify whether IGF2R is involved in regulating the autophagic activity induced by miR-352 inhibition, the ratio of LC3II to LC3I was measured in RACEs treated with miR-352 mimic, miR-352 inhibitor, siIGF2R or miR-352 inhibitor plus siIGF2R. We found that inhibition of miR-352 increased the LC3II/LC3I ratio, whereas miR-352 mimic reduced the LC3II/LC3I ratio (Figure S11). On the other hand, we found that the ratio of LC3II to LC3I was significantly lower in the RACEs treated with miR-352 inhibitor plus siIGF2R, as compared to those treated with miR-352 inhibitor (Figure S11), indicating that the miR-352-IGF2R pathway may regulate autophagy during collateral vessel growth.

## Discussion

The main findings of this study areas follows: (1) a different miRNA expression profile exists in collateral vessels induced by elevated FSS in the rat AV-shunt model; (2) miR-352 is down-regulated and has an anti-arteriogenic role in FSS-induced collateral vessel growth; (3) miR-352 regulates autophagy through regulating its target gene IGF2R/CI-M6P during arteriogenesis in a rat AV-shunt model.

The restoration of maximal conductance through the body’s natural repair mechanism in animals after arterial occlusion only reaches 35% to 40% of normal^21, 22^. However, high levels of FSS generated by shunting most of the collateral flow directly into the accompanying venous system could normalize the maximal conductance^7, 8^. In this AV-shunt model, the growth of collateral vessels was dramatically enhanced, not only in size but also in number, as compared with that in the ligature-only side^7, 8^. We previously demonstrated that several molecules, such as TRPV4 and ABRA, play an important role in this high FSS induced arteriogenic response^9, 23^ and identified several groups of differentially expressed mRNAs in this AV-shunt model^9^, implying that altered gene expression is involved in this rapid and amplified collateral vessel growth. Here, we revealed that miRNAs control this amplified collateral vessel growth by mediating gene expression, showing that there exists a differential miRNA expression profile of a total of 94 differentially expressed miRNAs with 44 up-regulated and 50 down-regulated miRNAs compared to the ligature-only side of the collaterals. Furthermore, among these miRNAs, many were found to be involved in angiogenesis, including several with a regulatory function in cell proliferation, the expression of growth factors and extracellular proteolysis, such as miR-497, miR-98, miR-181a, miR-145, miR-29b and miR-27a^24–29^. This is in agreement with our previous reports, which showed that these arteriogenic events are significantly augmented in the AV-side collateral vessels^7, 30^, implying that these miRNAs are likely novel candidates that regulate elevated FSS-induced collateral vessel growth.

In the miRNA expression profile, we found several miRNAs including miR-352, that have not been previously described as regulators in vascular biology. miR-352 was first found in E18 rat primary cortical neurons^31^. It is closely related to let-7d-5p (BLAST, blast.ncbi.nlm.nih.gov), which is also down-regulated by FSS, but does not share the same seed sequence. To determine whether miR-352 is a novel candidate that regulates collateral vessel growth, we used 3 bioinformatic algorithms (miRanda [www.microrna.org], miRDB [mirdb.org/miRDB] and targetscan [www.targetscan.org]) to screen potential miRNA seed sequences in the 3′-UTR and identified IGF2R, which has a potential miR-352 target site that was predicted by all bioinformatic algorithms. IGF2R, a multiple ligand-binding cell transmembrane glycoprotein that is ubiquitously expressed in mammal tissues, plays a fundamental role in regulation of autophagy, apoptosis and mitogenic effects^32, 33^. Moreover, our group previously demonstrated that IGF2R is up-regulated in collaterals by FSS in the rabbit model^9^. Its function in collateral vessel growth has not yet been investigated. All the above-mentioned findings indicate that miR-352 could be a novel miRNA regulator of elecated FSS-induced-collateral vessel growth. Indeed, this hypothesis was confirmed by the present study. We first used the *in situ*-hybridization approach to localize the cells that express miR-352 in collaterals and found that both, endothelial and smooth muscle cells express miR-352. Subsequently, we investigated the role of miR-352 in angiogenesis *in vitro* by modulating miR-352 levels with both, loss-of-function and gain-of-function approaches using cultured rat endothelial cells. We chose endothelial cells for the *in vitro* experiment since the endothelium is directly exposed to FSS and plays an important role in arteriogenesis^1^. We found that overexpression of miR-352 suppressed endothelial cell proliferation, migration and network formation, whereas inhibition of miR-352 promoted endothelial network formation, cell migration and proliferation, suggesting an anti-angiogenic potency of miR-352. Furthermore, using the *in vivo* antagomir approach, we demonstrated that inhibition of miR-352 promotes cell proliferation, collateral vessel growth and collateral flow restoration after femoral ligature in the rat hind limb. These data lead to the conclusion that miR-352 is not only an anti-angiogenic regulator but also an anti-arteriogenic regulator.

Analysis using 3 bioinformatic algorithms predicted that IGF2R is the target of miR-352. Indeed, we found that in AV-shunt collaterals down-regulation of miR-352 expression was accompanied by increased IGF2R expression. Moreover, an increase in IGF2R expression in collaterals was evident after miR-352 gene silencing. Furthermore, our *in vitro* experiments demonstrate that inhibition or overexpression of miR-352 results in the up-regulation or down-regulation of IGF2R expression in endothelial cells respectively. These findings confirm that IGF2R is negatively regulated by miR-352. In addition, we showed that knockdown of IGF2R suppresses cell proliferation and endothelial network formation *in vitro*, indicating that IGF2R plays an important role in cell proliferation and angiogenesis. This is in line with two previous reports that showed that IGF2R is required for angiogenesis^18, 19^. The function of IGF2R is initiated by binding to its cognate ligand IGF2, a protein hormone known to regulate cell proliferation, growth, migration, differentiation and survival^34^. We observed that increased expression of IGF2R was accompanied by up-regulation of IGF2 in AV-shunt side collateral vessels, suggesting that the IGF2-IGF2R pathway was involved. Taken together, these data demonstrate that the miR-352-IGF2R pathway is involved in angiogenesis and arteriogenesis.

In addition to its angiogenic potency, IGF2R is known to regulate autophagy, apoptosis and cell survival^33, 35^. Most recently, Tao *et al*. showed that miR-352 regulates autophagy following ischaemic stroke^20^. In the present study using Beclin1 and LC3 as markers of autophagy, we found that inhibition of IGF2R in cultured RAECs resulted in a decrease in autophagic activity. On the other hand, inhibition of miR-352 *in vivo* led to an up-regulation of IGF2R expression and increased autophagic activity in collaterals, which was indicated by increased expression of several autophagy-related genes, including Beclin1, ATG5 and LC3. Therefore, we propose that miR-352 regulates autophagy during collateral vessel growth by targeting IGF2R expression. Autophagy is a catabolic process that is important for protein degradation, organelle turnover and the recycling of cytoplasmic components, which allow cells to adapt to changing nutritional and energy demands by repurposing existing cellular components to sustain cellular metabolism, homeostasis, and survival^36^. Although the role of autophagy in the cardiovascular system is somehow controversial^37^, our data support the notion that increased autophagy facilitates collateral vessel growth. On one hand, endothelial cells activated by elevated FSS contain numerous cellular organelles; in particular, there are large numbers of free ribosomes in the cytoplasm^38–40^. They also exhibit increased expression of endothelial NO synthetase (eNOS), monocyte chemoattractant protein (MCP-1) and the adhesion molecules ICAM-1 and VCAM^1, 6, 40^. On the other hand, when endothelial cell autophagy was impaired by Atg3 siRNA or the autophagy inhibitor 3-MA, FSS was incapable of increasing eNOS expression, phosphorylation and NO production^41, 42^. Furthermore, Du *et al*. showed that induction of autophagy promotes angiogenesis while inhibition of autophagy suppresses angiogenesis^43^. It should be noted that the miR-352-IGF2R pathway may not be the only mechanism for regulating autophagy during collateral vessel growth because additional miRNAs involved in autophagy were identified, such as, miR-30. Pan *et al*. reported that miR-30-regulated autophagy mediates angiotensin II-induced myocardial hypertrophy^44^, which implies that there exists a complicated network for mediating autophagy that has yet to be determined.

In conclusion, we have demonstrated that differential expression of numerous miRNAs might be an important mechanism for rapid and augmented collateral vessel growth in the AV-shunt model. We identified that miR-352 is a novel candidate that negatively regulates collateral vessel growth and demonstrated that miR-352 is an endogenous IGF2R modulator in the regulation of angiogenesis and autophagy. However, it should be noted that although miRNAs-including miR-352 in this paper and other miRNAs found in previous studies, such as miR-100, miR-155, miR-329, and miR-495- are important factors controlling FSS-induced collateral vessel growth^15–17^, the mechanism by which miRNAs expression is regulated remains unclear. Currently there is no evidence regarding the interaction between FSS and the expression of those miRNAs, therefore, further elucidation of how miRNAs are regulated is needed and may lead to clinical applications of miRNAs as therapeutic tools.

## Materials and Methods

### Animal model

The experimental protocol was approved by the Animal Care and Use Committee of Central South University and adhered to the Guidance Suggestions for the Care and Use of Laboratory Animals by the Ministry of Science and Technology of the People’s Republic of China. Adult Sprague-Dawley (SD) rats (250–300 g, Animal Center, Xiangya School of Medicine, Central South University, Changsha, China) were anaesthetized with chloral hydrate (300 mg/kg, intraperitoneally). Bilateral femoral artery occlusion was performed in animals by ligating the femoral artery. On the left side, an arterio-venous (AV) fistula was created side-to-side between the distal femoral artery stump and the accompanying femoral vein, and the right side was used as a control^8^. Then, the skin was closed with sterile surgical clips. The animals were allowed to recover completely and were housed with free access to water and food. Gangrene and gross impairment of hind limb function were not observed in any of the rats. A total of 15 SD rats were used for the AV model.

### Tissue preparation

For immunofluorescence staining, the animals were killed under anaesthesia one week after the operation. Quadricep and adductor muscles were carefully removed for the experiments. Base on our previous experience, these muscles contain one or two collateral vessels during arteriogenesis. A total of 36 vessels were investigated. All samples were immediately frozen in liquid nitrogen, embedded in tissue processing medium (Tissue-Tek O.C.T, Sakura, CA, USA), and stored at −80 °C until further analysis.

To isolate collateral arteries, the hind limb vasculature was visualized with liquid latex as previously described^45^. Animals were anaesthetized, the aorta was cannulated, and the animals were exsanguinated then warm (37 °C) liquid latex (THAI HUA TEX, Thailand) was infused at a pressure of 80 mmHg. Collateral arteries bypassing the site of the operation were carefully dissected from the quadricep and adductor muscles after the hardening of the latex under a stereomicroscope and snap frozen in liquid nitrogen until RNA isolation. In addition, adductor muscles were harvested and used as positive controls for RT-qPCR analysis of troponin I1 (Tnni1) and troponin I2 (Tnni2) expression.

### miRNA microarray analysis

Total small RNA was isolated from collateral arteries in the quadricep and adductor muscles from 5 rats using the by mirVana^tm^ miRNA Isolation Kit (Ambion, Life Technologies, CA, USA) at one week after the operation. A portion of RNA from each sample was pooled in equal amounts, and microarray analysis for all identified rat miRNAs (miRBase 16.0) was performed by the Exiqon miRNA Expression Profiling service (Kangchen Bio-tech, China). The other portion of RNA in each sample was used for further qPCR analysis of miRNA expression.

### ***In vivo*** antagomir

Following the occlusion, 5 SD rats underwent intra-arterial injection of 500 nmol/kg antagomir (Ribobio, China) via subcutaneously implanted osmotic mini pumps (Model 1007, Alzet® Osmotic Pumps, CA, USA) connected to a catheter. The catheter was inserted retrograde into the proximal region of the occluded femoral artery. An antagomir-control was used as a negative control.

### Postmortem angiograms

Postmortem angiography was performed by injecting the contrast medium as previously described^22^. Gelatine (20 g, type A, Sigma, MO, USA) was dissolved in 100 ml of 50 °C distilled water with continuous steering. Afterward, 100 g of lead oxide (Sigma, MO, USA) was added and mixed well. Animals were anaesthesized and anticoagulated, and the aorta was cannulated. Subsequently, the animals were bled and immersed in water warmed to 50 °C. Then, the contrast medium was infused at a pressure of 150–180 mmHg until filling of the distal femoral stump was observed. The animals were immediately placed on crushed ice, and the contrast medium was allowed to harden under continuous pressure. X-ray images were taken in an X-ray chamber (model DHF-155H, Tokyo Japan, HITACHI MEDICAL CORPORATION) exposed to 50 KV and 100 mA for 30 mS. Images were acquired and transferred to a computer, which was used to identify and quantify the collateral arteries.

### Collateral flow measurements

Collateral flow measurements were performed as previously described^46, 47^. Briefly, at 7 days after the initial operation and antagomir treatment, the animals were anaesthetized with chloral hydrate (300 mg/kg, intraperitoneally). A catheter was inserted in the abdominal aorta with the tip just proximal to the aortic bifurcation. Both hind limbs were perfused at 3 different pressures (60, 80 and 100 mm Hg) with heparinized saline and adenosine (1.0 mg/kg per min, Sigma, MO, USA) to achieve maximal vasodilatation. At each pressure level, 3 × 10^6^ red microspheres of 15 μm in diameter (Molecular Probes, OR, USA) were thoroughly mixed and injected into the perfusion system. At the distal end of the perfusion system, a reference blood sample was withdrawn for one minute at a rate of 0.4 mL/min after the onset of microsphere injection.

After the microsphere injection, the animals were euthanized, and their hind limb muscles (i.e., quadricep and adductor muscles) were dissected. Tissue was digested and homogenized with SDS/proteinase solution for FACS analysis and microsphere quantification. Restoration of flow was then expressed as a percentage derived from the ratio between the flows in the experimental hind limb versus that in the non-occluded hind limb.

In addition, histological studies of the tissues perfused with fluorescence microspheres were performed. Quadricep and adductor muscles were carefully removed under anaesthesia after microsphere injection and embedded in tissue processing medium. Cryosections 20 μm thick were obtained and fixed in 4% paraformaldehyde. Then sections were incubated with Actin-Phalloidin (FITC labelled), cover-slipped and viewed with a Nikon confocal microscope (Nikon, Japan).

### Statistical analysis

All data were presented as the mean ± SEM using Prism 5 (GraphPad Software Inc, CA, USA). Treatment groups were compared by unpaired Student’s t-test, and one-way ANOVA was used for multiple comparisons of >2 groups. A *P* value < 0.05 was considered statistically significant.

## Electronic supplementary material

Supplementary material
