# Supplementary material for: microRNA-352 regulates collateral vessel growth induced by elevated fluid shear stress in the rat hind limb
Source: Sci Rep. 2017 Jul 27;7:6643. doi: 10.1038/s41598-017-06910-9 (PMC5532297; doi:10.1038/s41598-017-06910-9)
Supplement: Supplementary file 1 — Supplementary material [file 41598_2017_6910_MOESM1_ESM.pdf]

# microRNA-352 regulates collateral vessel growth induced by elevated fluid shear stress in the rat hind limb

Yinglu Guan[1,6], Baizhen Cai[2], Xiaoqiong Wu[3], Song Peng[1,4], Liaoying Gan[1], Da Huang[1], Guangmin Liu[1], Liping Dong[1], Lin Xiao[1], Junwen Liu[1], Bin Zhang[1], Wei-Jun Cai[1], Jutta Schaper[5] & Wolfgang Schaper[5]

[1]Department of Histology & Embryology, School of Basic Medicine, Central South Universitiy Changsha，410013, Hunan, P.R. China; [2]Departmet of Intensive Care Unit, the 3rd Xiangya Hospital, Central South University Changsha, 410013, Hunan, P.R. China; [3]Department of Anatomy & Neurobiology, School of Basic Medicine, Central South University Changsha, 410013, Hunan, P.R. China; [4]Department of Radiology, the 3rd Xiangya Hospital, Central South University Changsha, 410078, Hunan, [5]Max-Planck-Institute for Heart and Lung Research, Arteriogenesis Research Group, Bad Nauheim, D-61231, Germany; [6]Department of Pharmacological & Pharmaceutical Sciences, College of Pharmacy, University of Houston, 77204, Houston, TX, US

Corresponding author:

Bin Zhang, M.D., Ph.D. 172 Tong-Zhi-Po Road, Changsha, 410013, P.R.China

E-mail: coolzhangbin22@163.com

Jutta Schaper, M.D., Ph.D. Parkstr. 1 D-61231 Bad Nauheim, Germany

Tel: ++49-6032/705-409 Fax: ++49-6032/705-458

E-mail: jutta.schaper@mpi-bn.mpg.de

Wei-Jun Cai, M.D., Ph.D. 172 Tong-Zhi-Po Road, Changsha, 410013, P.R.China

E-mail: caiweijun@csu.edu.cn / wjcai@yahoo.com

## SUPPLEMENTAL MATERIAL

A) Supplementary methods

B) Supplementary figures

1. Diagram showing the arterio-venous shunt.
2. Hierarchical clustering for normalized data of miRNAs in all samples.
3. qPCR analysis of Tnni1 and Tnni2 in collateral vessel samples.
4. miR-352 target genes predicted by all three bioinformatic algorithms.
5. Representative micrograph of CD31 and mimic negative control in the RAECs.
6. miR-352 expression in cultured RAECs.
7. The role of miR-352 in endothelial cell viability and proliferation.
8. Diagram showing in vivo application of antagomir-352 via osmotic mini pumps and its effect on miR-352 expression in collateral vessels.
9. qPCR showing the effect of siIGF2R on IGF2R expression in RAECs.
10. qPCR assay of autophagic marker expression, including LC3a, LC3b and ATG5, in collateral vessels of the shunt or antagomir-352 treated rats.
11. Western blot analysis of the LC3II/LC3I ratio in RACEs treated with miR-352 mimic, miR-352 inhibitor, siIGF2R, or miR-352 inhibitor plus siIGF2R.

C) Supplementary tables

1. microRNA microarray results
2. Antibodies
3. TaqMan Assay and PCR primers
4. In situ probes for microRNA
5. Transfected sequences

## Supplementary methods

**Immunofluorescence.** Cryosections were cut at 5 μm thickness and fixed in 4% paraformaldehyde. Following incubation in 0.5% BSA (Solarbio, China) with 0.3% Triton X-100 (SigmaAldrich, MO, USA), the sections were incubated with primary and secondary antibodies (Table S2), followed by Cy2- or Cy3-conjugated streptavidin (Biotrend, Germany). The nuclei were stained with 4',6-diamidino-phenylidole (DAPI, Invitrogen, Life Technologies, OR, USA). The sections were coverslipped and viewed with a Nikon confocal microscope (Nikon, Japan).

Immunostaining of cultured cells was performed following a similar protocol as that described above, except the primary antibody was incubated at 4°C overnight.

Incubation with phosphate-buffered saline (PBS) instead of the primary antibody was performed as negative control to exclude nonspecific binding of the secondary detection system.

**Quantitative measurements.** Quantification of the immunofluorescence intensity of IGF2R, IGF2 and Beclin1 was performed with a Nikon confocal microscope, using the quantitation software EZ-C1 3.70. Briefly, one channel with format 512 and appropriate filters were used. A full range of grey values from black to peak white (0-pixel to 255-pixel intensity level) was set during the entire measurement process. Fluorescence intensity of was expressed as arbitrary units (AU/$\mu m^2$).

Quantitative analysis of Ki67 was performed using the confocal microscope. Quantification was performed at 40×, and the ratio of the number of Ki67 positive nuclei to the total number of nuclei in the vascular wall was considered as the proliferation index.

**RT-PCR.** Total small RNA was isolated using the mirVana$^{tm}$ miRNA Isolation Kit (Ambion, Life Technologies, CA, USA), and miRNA expression was validated by quantitative stem-loop PCR technology (TaqMan microRNA Assays, Applied Biosystems, Life Technologies, CA, USA) as previously described [1] (Table S3). The use of target-specific reverse transcription primers and TaqMan hybridization probes allows the specific detection of mature miRNAs. miRNA expression was normalized to the expression of U6.

Total RNA was isolated using TRIzol (Invitrogen, Life Technologies, CA, USA) according to the manufacturer's protocol. Afterwards, 1 μg of total RNA was transcribed to cDNA (Fermentas, Thermo Scientific, Lithuania), and real-time PCR was performed on a mastercycler nexus (Eppendorf, Germany). The expression of 18s RNA served as a loading control. Primer sequences are listed in Table S3.

**microRNA in situ hybridization.** In situ hybridization was performed on 10μm frozen tissue sections using locked-nucleic acid probes (Exiqon, Denmark) based on a modified version of the protocol described by Heller et al[2]. Sections were thawed for 20 min at room temperature, incubated with protein kinase K (Merck, MA, USA) for 20 min at 37°C, washed for 10 min with PBS and fixed with 4% paraformaldehyde for 10 min. After washing 3 times with PBS, sections were acetylated for 10 min in triethanolamine (Kermel, China) and acetic anhydride (Huihong, China) and washed once. Sections were incubated with

hybrization buffer for 1 h at room temperature; meanwhile, probes (3'and/ or 5'-DIG labelled LNA probes) were mixed with hybridization buffer, heated to 65°C for 5 min, chilled on ice, added to the sections incubated overnight at 54°C. After incubation for 1 h in 50% fomamide/ 1×SSC at 54°C, 15 min in 0.2× SSC at room temperature and washed for 15 min in PBS. Sections were blocked with blocking reagent at room temperature for 1 h. For the localization of miR-352 expression in endothelial cells, following anti-DIG-FITC (Roche, Germany) incubation and a wash, the sections were incubated with CD31 (marker of endothelial cells) and secondary antibodies (Table S2), followed by Cy3-conjugated streptavidin (Biotrend, Germany). For the localization of miR-352 expression in smooth muscle cells, anti-DIG-FITC and α-smooth muscle actin Cy3-conjugated were added for 1h at 37°C. Sections were washed 3 times in PBS with 0.1% Tween-20 (Sigma Aldrich, MO, USA) and 2 times in PBS; and then, they were mounted in DAPI mounting medium. U6 was used as a positive control (Table S4).

**Cell culture.** Rat aorta endothelial cells (RAECs) and endothelial basal medium were purchased from Chiscinentific (CHI Scientific, China) and cultured until the fifth passage.

**Transfection.** For transfection with miRNA mimic or miRNA inhibitor (Ribobio, China), RAECs were cultured to 70% confluence before transfection. For overexpression of miR-352, RAECs were transfected with 50 nM miR-352 mimic or mimic ncontrol using Lipofectamine 2000 (Invitrogen, Life Technologies, CA, USA) according to the manufacturer's protocol. For inhibition of miR-352, RAECs were transfected with 100 nM miR-352 inhibitor or miRNA inhibitor Ncontrol using Lipofectamin 2000. For sequences, see Table S5.

For siRNA-mediated gene knockdown, RAECs were cultured to 70% confluence and transfected with IGF2R siRNA (Ribobio, China) and miRNA inhibitor. A scrambled siRNA was used as a control.

**Luciferase reporter assay.** The 1257 bp IGF2R-3'UTR fragment includes a conserved binding site for miR-352. To examine whether miR-352 transcription occurs through IGF2R, RAECs were transfected with miR-352 mimic and miRNA Target IGF2R-3'UTR Vector Plasmid or mutant plasmid (Ribobio, China) in the same final concentrations using Lipofectamine 2000 as stated above (for sequences, see Table S5). At 48 h post-transfection, cell lysates were collected, and the activities of firefly and renilla luciferase were assessed with the Dual-Luciferase Reporter assay system (Promega, WI, USA).

**Western blot.** For Western blot analysis of IGF2R expression, RAECs were lysed in RIPA buffer (CWBio, China) for 20 min on ice. After centrifugation for 10 min at 14,000×g (4°C), the protein content of the samples was quantified using the BCA method. Equal amounts of protein were separated by SDS-PAGE and blotted onto nitrocellulose membranes. Western blots were incubated overnight with antibodies directed against IGF2R; β-actin (CWBio, China) was used as a loading control.

**Tube formation assay.** At 48 h after transfection, RAECs ($3\times10^4$ cells) were cultured

in a 48-well plate (Corning, NY, USA) coated with 100 μl of Matrigel Basement Membrane Matrix (Corning, MA, USA) for 6 h. Images were acquired at 5 randomly chosen microscopic fields. The cumulative tube length and the number of branch points were analysed with AxioVision 4.6 (Zeiss, Germany).

**MTT viability assay.** Assessment of cellular viability was performed at 24h following transfection by measuring the metabolization of MTT [3-(4,5-dimethylthiazol-2-yl)-2,5-diphenyl-2H-tetrazolium bromide] (Solarbio, China). MTT (1 mg/ml) was added to each well and the cells were cultured for an additional 4 h or 24 h, washed with PBS and lysed in dimethyl sulfoxide (DMSO, Solarbio, China). Absorbance was photometrically measured at a wavelength of 490 nm.

**Migration assay.** To evaluate the migration ability of RAECs, the cells were detached with trypsin (0.25% EDTA), harvested by centrifugation at 1,000×g, resuspended in 500 μl of EBM with 1% BSA, counted and placed in the upper chamber of Transwell chambers ($5\times10^4$ cells per chamber, pore size 8 μm, Corning, ME, USA). The chamber was placed in a 24-well culture dish containing complete EBM. After incubation for 48 h, the non-migrated cells on the upper side of the chamber were mechanically removed and the remaining cells on the lower side were fixed with 95% alcohol. For quantification, cells were stained with crystal violet (Kermel, China). Stained migrated cells on the bottom side of the chamber were lysed in 33% acetic acid. Optical density was measured at 570 nm.

## References

1. Varkonyi-Gasic, E., Wu, R., Wood, M., Walton, E.F. & Hellens, R.P. Protocol: a highly sensitive RT-PCR method for detection and quantification of microRNAs. *Plant methods* **3**, 12 (2007).
2. Heller, S., Sheane, C.A., Javed, Z. & Hudspeth, A.J. Molecular markers for cell types of the inner ear and candidate genes for hearing disorders. *Proceedings of the National Academy of Sciences of the United States of America* **95**, 11400-11405 (1998).

## Supplementary figures

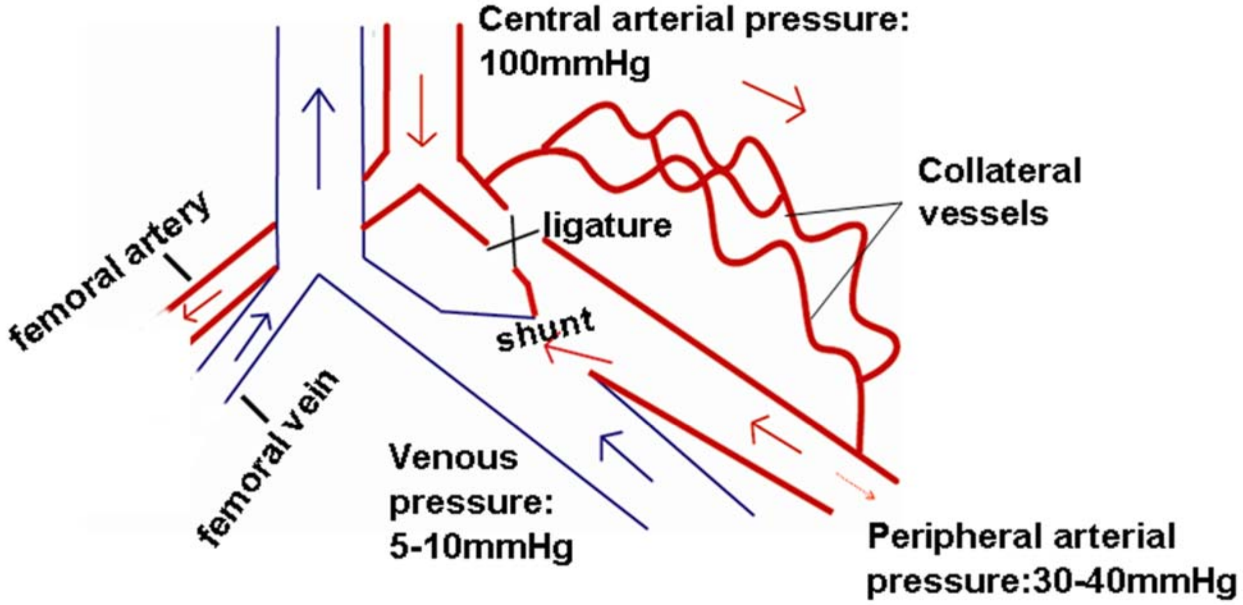


**Figure S1:** Diagram showing the arterio-venous shunt. An arterio-venous shunt was created between the distal stump of the occluded femoral artery and vein. Because of the steep blood pressure gradient along the collateral arteries (arterial pressure at the collateral stem and venous pressure at re-entry), collateral blood flow is increased. Fluid-shear-stress along the collateral artery becomes maximal.

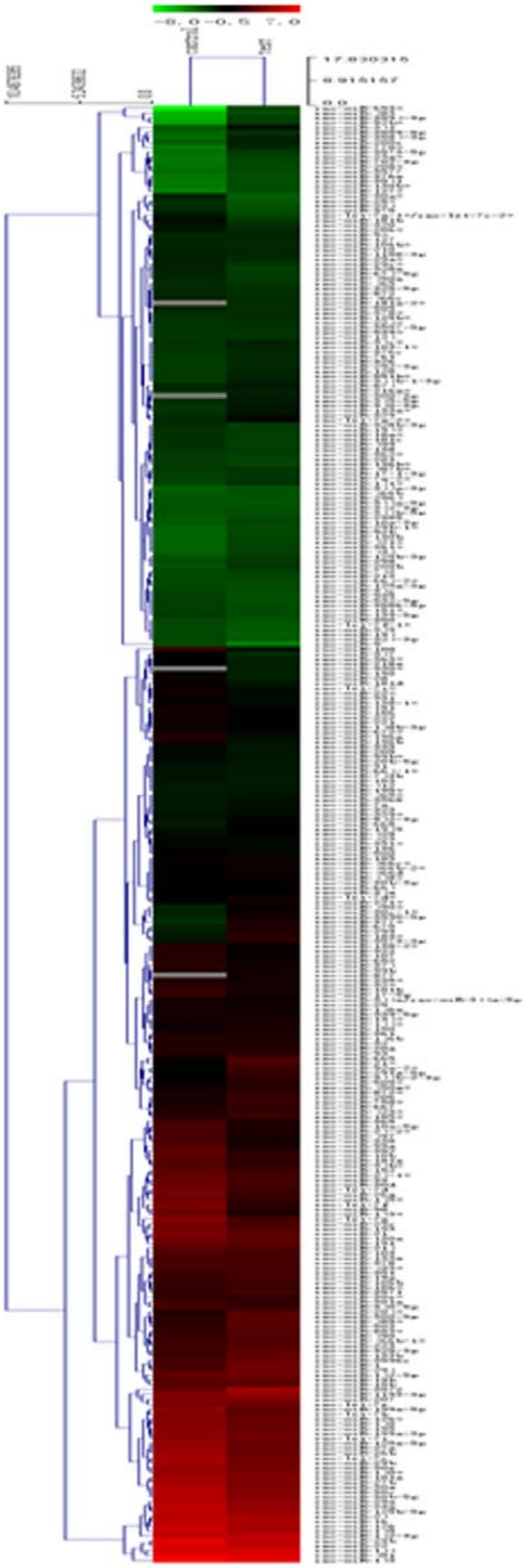

**Figure S2:** Hierarchical clustering for normalized data of miRNAs in all samples. Red indicates high relative expression, and green indicates low relative expression.

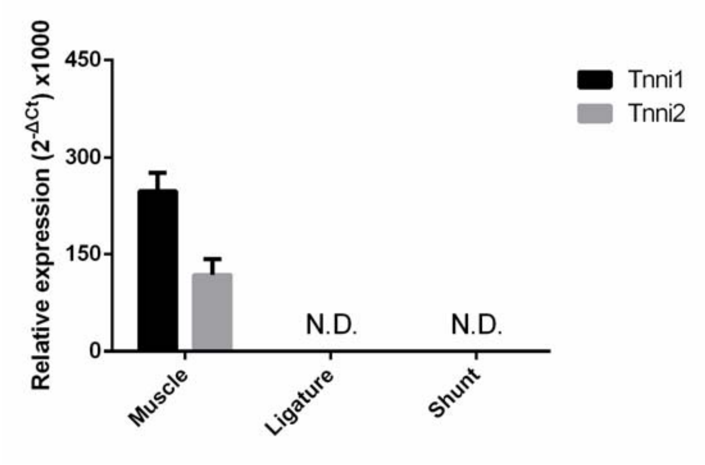


**Figure S3:** qPCR analysis of Tnni1 and Tnni2 in collateral vessel samples

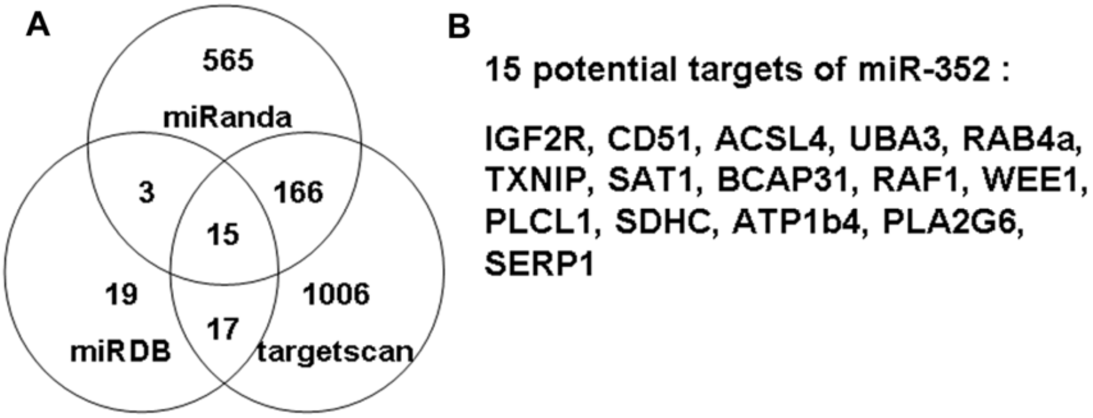


**Figure S4:** miR-352 target genes predicted by all three bioinformatic algorithms. A: Three bioinformatic algorithms were applied; B: the 15 targeted genes.

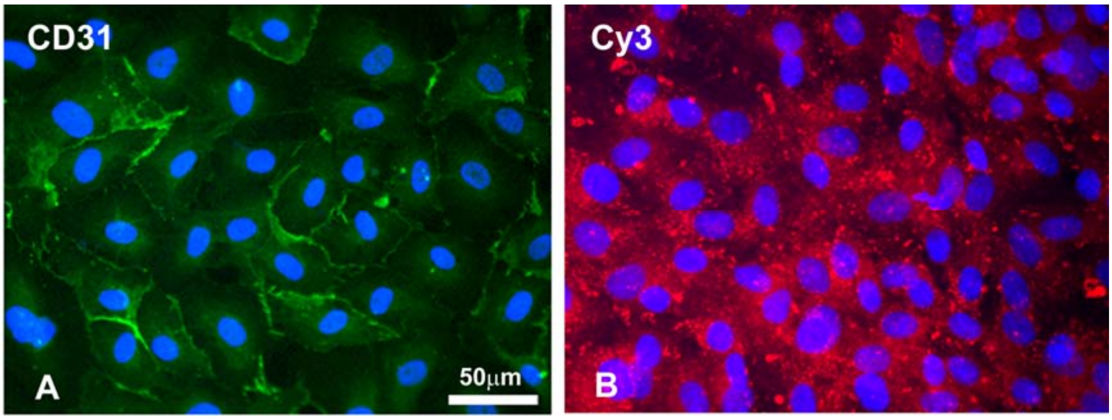


**Figure S5:** Representative micrographs of CD31 (A) and mimic negative control (B) in the RAECs.

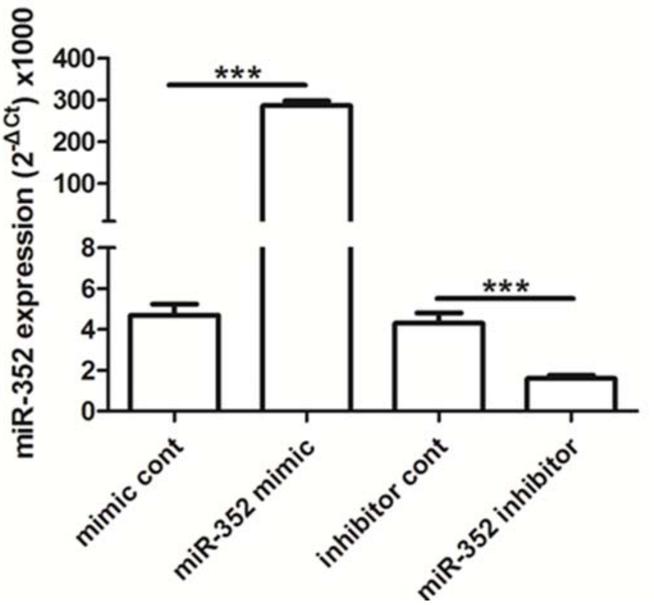


**Figure S6:** miR-352 expression in cultured RAECs.

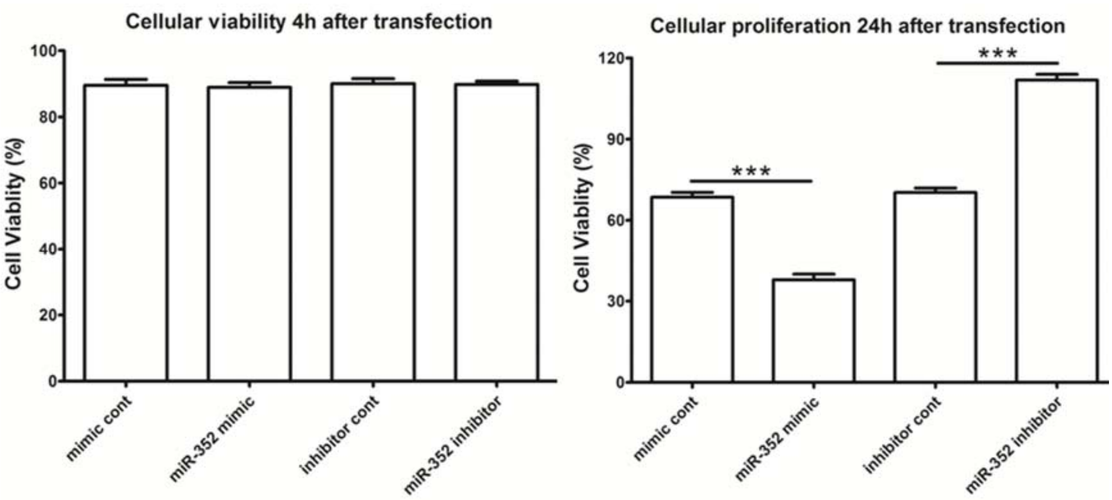


**Figure S7:** The role of miR-352 in endothelial cell viability and proliferation.

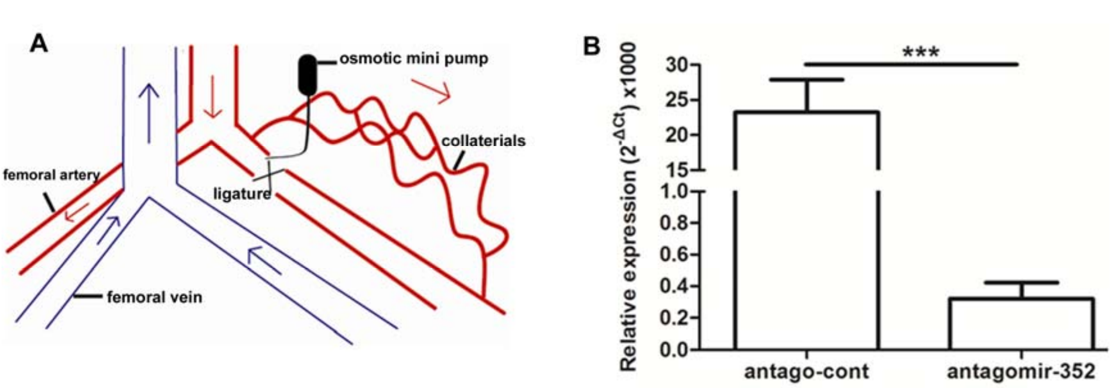


**Figure S8:** Diagram showing the in vivo application of antagomir-352 via osmotic mini pumps and its effect on miR-352 expression in collateral vessels.

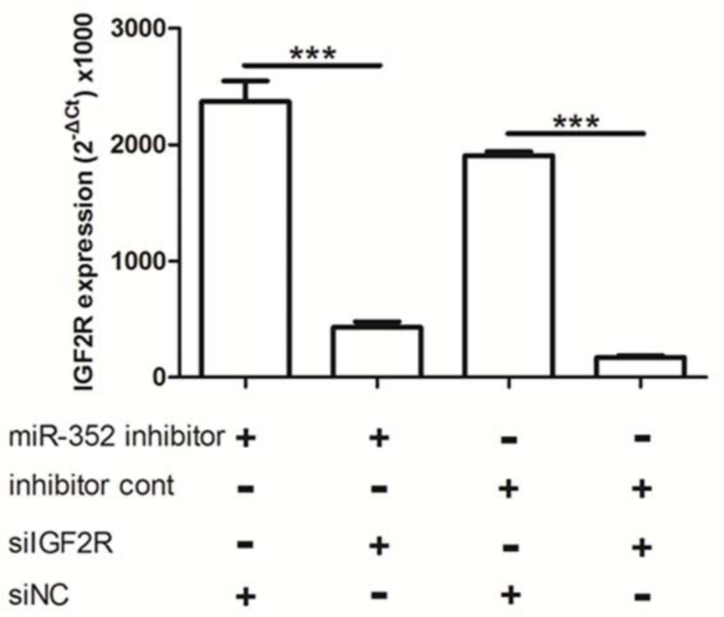


**Figure S9:** qPCR showing the effect of siIGF2R on IGF2R expression in RAECs

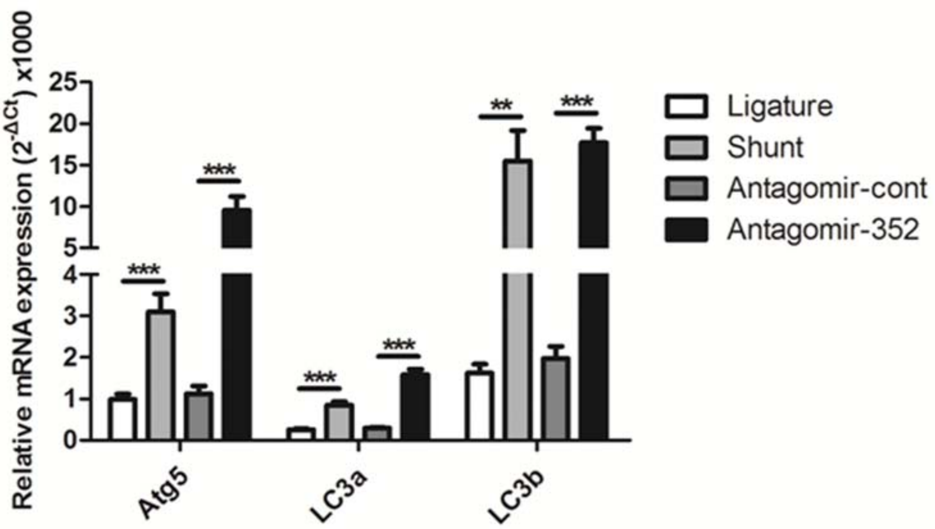


**Figure S10:** qPCR assay of expression of autophagic markers, LC3a, LC3b and ATG5 collateral vessels in shunt or antagomir-352 treated rats

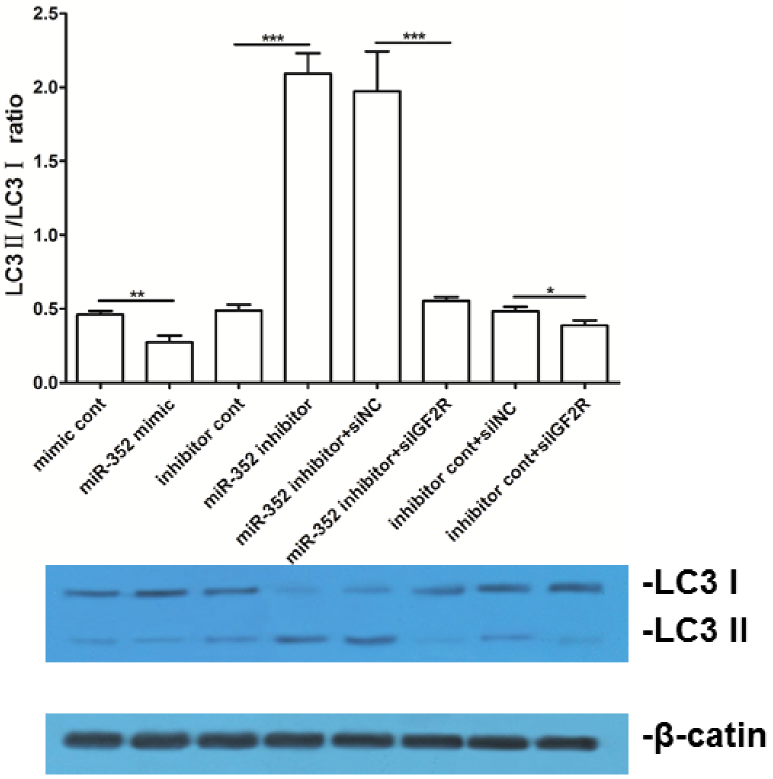


**Figure S11:** Western blot analysis of the LC3II/LC3I ratio in RACEs treated with miR-352 mimic, miR-352 inhibitor, siIGF2R, or miR-352 inhibitor plus siIGF2R.

## Supplementary tables

**Table S1:** microRNA microarray results

| Up-regulated | Fold change AV-shunt vs. control | Down-regulated | Fold change AV-shunt vs. control |
|---|---|---|---|
| rno-miR-653-3p | 37.56097561 | rno-let-7f-5p | -5.200726271 |
| rno-miR-3594-3p | 9.31358885 | rno-miR-98-5p | -4.790951973 |
| rno-miR-483-3p | 8.414634146 | rno-miR-100-5p | -4.56496786 |
| rno-miR-92b-5p | 8.096126255 | rno-miR-143-5p | -4.399502269 |
| rno-miR-770-3p | 5.407129456 | rno-miR-30a-3p | -3.848484848 |
| rno-miR-377-5p | 4.84735544 | rno-let-7a-5p | -3.643793535 |
| rno-miR-341 | 4.764878049 | rno-miR-26a-5p | -3.478532104 |
| rno-miR-21-3p | 4.637785208 | rno-miR-145-3p | -3.171432554 |
| rno-miR-3584-3p | 4.307588076 | rno-miR-99a-5p | -3.150019751 |
| rno-miR-675-5p | 4.143757751 | rno-let-7a-1-3p/rno-let-7c-2-3p | -2.983700643 |
| rno-miR-92a-2-5p | 3.808690916 | rno-miR-497-5p | -2.888349061 |
| rno-miR-183-3p | 3.74730108 | rno-miR-9a-5p | -2.880165289 |
| rno-miR-299a-5p | 3.626723224 | rno-miR-352 | -2.873047456 |
| rno-miR-200c-3p | 3.593610443 | rno-let-7d-5p | -2.854490731 |
| rno-miR-665 | 3.511737089 | rno-miR-674-5p | -2.863621876 |
| rno-miR-291a-5p | 3.457928187 | rno-miR-28-5p | -2.83042344 |
| rno-miR-3572 | 3.445658165 | rno-miR-29b-3p | -2.78013317 |
| rno-miR-3573-3p | 3.261484363 | rno-miR-347 | -2.776367022 |
| rno-miR-3590-5p | 3.227251407 | rno-miR-30c-5p | -2.774625087 |
| rno-miR-3573-5p | 3.097030753 | rno-let-7c-5p | -2.738042055 |
| rno-miR-300-5p | 2.87653263 | rno-miR-338-3p | -2.725543031 |
| rno-miR-23a-5p | 2.866151101 | rno-miR-30a-5p | -2.712940006 |
| rno-miR-485-3p | 2.803583594 | rno-miR-27a-3p | -2.699275761 |
| rno-miR-127-5p | 2.780487805 | rno-miR-26b-5p | -2.649331845 |
| rno-miR-3559-5p | 2.772357724 | rno-miR-196a-5p | -2.590468443 |
| rno-miR-30c-1-3p | 2.707609756 | rno-miR-10b-5p | -2.580960549 |
| rno-miR-702-3p | 2.644599303 | rno-miR-196b-5p | -2.572727273 |
| rno-miR-204-3p | 2.629048381 | rno-miR-29c-3p | -2.570647336 |
| rno-miR-130b-5p | 2.515243902 | rno-miR-199a-5p | -2.56509734 |
| rno-miR-1-3p | 2.505729499 | rno-miR-125a-5p | -2.54172347 |
| rno-miR-344b-2-3p | 2.470711715 | rno-miR-23a-3p | -2.495616162 |
| rno-miR-3541 | 2.454878049 | rno-miR-181b-5p | -2.423409923 |
| rno-miR-490-5p | 2.373358349 | rno-miR-28-3p | -2.420138889 |
| rno-miR-503-5p | 2.32738266 | rno-miR-24-2-5p | -2.39063622 |
| rno-miR-208-5p | 2.309650053 | rno-miR-27b-3p | -2.37321744 |
| rno-miR-133b-3p | 2.192278644 | rno-miR-10a-5p | -2.361033011 |
| rno-miR-376a-3p | 2.159428911 | rno-miR-30b-5p | -2.305271642 |
| rno-let-7a-2-3p | 2.086275937 | rno-miR-125b-5p | -2.213879771 |
| rno-miR-883-5p | 2.021797788 | rno-miR-145-5p | -2.209689889 |

| | | | |
|---|---|---|---|
| rno-miR-329-3p | 2.021238696 | rno-miR-224-5p | -2.198135198 |
| rno-miR-434-3p | 2.018792483 | rno-miR-190a-5p | -2.170155502 |
| rno-miR-3577 | 2.012195122 | rno-miR-181a-5p | -2.160005478 |
| rno-miR-294 | 2.010495633 | rno-miR-30d-5p | -2.153802865 |
| rno-miR-3596c | 2.005032907 | rno-miR-30e-5p | -2.152844478 |
| | | rno-miR-33-5p | -2.148591392 |
| | | rno-let-7b-5p | -2.146867493 |
| | | rno-miR-23b-3p | -2.135993695 |
| | | rno-miR-375-3p | -2.070707071 |
| | | rno-miR-195-5p | -2.042363996 |
| | | rno-miR-34b-3p | -2.023000868 |

**Table S2:** Antibodies

| Primary antibody | Host species | Clone ID | Company | label |
|---|---|---|---|---|
| IGF2R | mouse | 2G11 | Abcam, UK | - |
| IGF2 | rabbit | - | Abcam, UK | - |
| Ki67 | rabbit | SP6 | Vector Lab, CA, USA | - |
| Beclin1 | rabbit | - | Abcam, UK | - |
| LC3B | rabbit | D11 | Cell Signaling Technology, MA, USA | - |
| CD31 | rabbit | EP3095 | Millipore, MA, USA | - |
| β-actin | mouse | 3E8 | CWbio, China | - |
| Actin-Phalloidin | - | - | Sigma Aldrich, MO, USA | TRITC |
| Actin-Phalloidin | - | - | Sigma Aldrich, MO, USA | FITC |
| SMA | mouse | 1A4 | Sigma Aldrich, MO, USA | Cy3 |

| Secondary antibody | Host species | Cat# | Company | label |
|---|---|---|---|---|
| mouse IgG | rabbit | 610-404-040 | Biotrend, Germany | Cy3 |
| rabbit IgG | donkey | 611-704-127 | Biotrend, Germany | Cy3 |
| mouse IgG | goat | BA-9200 | Vector Lab, CA, USA | biotin |
| mouse IgG | donkey | AP192P | Chemicon, Germany | HRP |
| rabbit IgG | goat | BA-100 | Vector Lab, CA, USA | biotin |
| rabbit IgG | donkey | AP182P | Chemicon, Germany | HRP |

**Table S3:**

TaqMan assay (Applied Biosystems, CA, USA)

| Assay name | Assay ID | Company |
|---|---|---|
| rno-mir-352 | 001339 | Applied Biosystems |
| U6 snRNA | 001973 | Applied Biosystems |

PCR-Primers (BGI, China)

| Gene | Forward | Reverse | Size(bp) |
|---|---|---|---|
| IGF2R | CACTGTGACCCTCTGCTGAA | CCAGAGGACACACAGCTGAA | 103 |
| IGF2 | TAACACCGGCTAGAGCCATCAAC | ACTCAGGCCAAGCGATAGAGACA | 102 |
| LC3a | CCAGCACAGCATGGTGAGTGTATC | AGAAGCCGAAGGTTTCTTGGGAG | 113 |
| LC3b | TGAATGGGCACAGCATGGTGAGT | CGAACGTCTCCTGGGAGGCATAG | 111 |
| ATG5 | AGGCTCAGTGGAGGCAACAG | CCCTATCTCCCATGGAATCTTCT | 72 |
| Beclin1 | CAAATGAATGAGGGCGACAG | TTCTCCAGGTTTTCTGCCAC | 131 |
| Tnni1 | GACCTGAAGCTGAAGGTGCT | TGGACACCTTGTGTTTGGAA | 121 |
| Tnni2 | GTGCAGGAACTCTGCAAACA | CAGCTCCTTGCTGCTCTTCT | 99 |
| RNA 18S | GTAACCCGTTGAACCCCATT | CCATCCAATCGGTAGTAGCG | 151 |

**Table S4**: In situ probes (Exiqon, Denmark)

| In situ probes | Label | No. | Company |
|---|---|---|---|
| miR-352 | 5’-DIG and 3’-DIG | 41165-15 | Exiqon |
| miR-159 | 5-DIG | 99003-01 | Exiqon |
| U6 | 5’-DIG | 99002-01 | Exiqon |

**Table S5**:

miRNA (Ribobio, China)

| miRNA | Cat # | Company |
|---|---|---|
| miR-352 mimic | miR10000610 | Ribobio |
| mimic Ncontrol | miR01101 | Ribobio |
| miR-352 inhibitor | miR20000610 | Ribobio |
| inhibitor Ncontrol | miR02101 | Ribobio |

siRNA (Ribobio, China)

| siRNA | Cat # | Company |
|---|---|---|
| si-r-IGF2R | siR1505181136 | Ribobio |
| NControl | siN05815122147 | Ribobio |
